# Considerations about the Brazilian Journal of Cardiovascular Surgery editorial profile

**DOI:** 10.21470/1678-9741-2017-0504

**Published:** 2017

**Authors:** Domingo M. Braile

**Affiliations:** 1 Editor-in-Chief - BJCVS

Dear reader,

Behold, another issue of Brazilian Journal of Cardiovascular Surgery (BJCVS) comes to you and we hope to contribute as a research and knowledge tool.

The good news we bring is that the BJCVS achieved a higher rating in the Impact Factor (IF), reaching the 0.601 index. It is an achievement of all of us, readers, authors, reviewers and members of the Editorial Board, who always believe and work for the scientific development of our country. This time, all the Brazilian journals have raised the IF, which shows that despite the political and economic crisis that we are facing, science is still able to survive.

In this edition, we bring 11 Original Articles, 1 Review Article and 1 Case Report dealing with current and relevant topics in the specialty, of national and foreign authorship.

A general analysis of this edition allows some general inferences that are surely associated with the positive evolution of our IF. Of the 13 published articles, 6 are originate in other countries. This fact is significant in relation to the search for the much desired "internationalization". It is worth noting that one of the articles that evaluate the protective action of the taladafil against the development of multiple organ failure syndrome, has as senior author Professor Ferid Murad, who shared with professors Robert Furchgott and Louis Ignarro the Nobel Prize for the discovery of nitric oxide.

Two articles, in essence, can be classified as translational by analyzing the presence of an antibiotic in mediastinal fat, the coronary pressure of the cardioplegic solution infusion, and the cardiac autonomic nervous system recovery after heart transplant.

Three articles exemplify our Society's constant concern with epidemiological aspects of surgical cardiopathies in Brazil: the ASSIST Consortium for Congenital Heart Defects and two trials related to ventilatory assistance, which reaffirm the valuable contribution of physiotherapy professionals.

Finally, two comments deserve mention to conclude this editorial. An optimist related to collaborations of pediatric surgery and maintenance of pessimism related to the low adhesion of adult cardiac surgery.

Have a great reading.

Kind regards,

**Domingo M. Braile** ^1^Editor-in-Chief - BJCVS

